# Institutional trust and alcohol consumption in Sweden: The Swedish National Public Health Survey 2006

**DOI:** 10.1186/1471-2458-8-283

**Published:** 2008-08-13

**Authors:** Johanna Ahnquist, Martin Lindström, Sarah P Wamala

**Affiliations:** 1 Swedish National Institute of Public Health, Stockholm, Sweden; 2 Karolinska Institutet, Stockholm, Sweden; 3 Department of Clinical Sciences, Malmö University Hospital, Lund University, Lund, Sweden; 4 Stockholm Centre of Public Health, Stockholm, Sweden

## Abstract

**Background:**

Trust as a measure of social capital has been documented to be associated with health. Mediating factors for this association are not well investigated. Harmful alcohol consumption is believed to be one of the mediating factors. We hypothesized that low social capital defined as low institutional trust is associated with harmful alcohol consumption.

**Methods:**

Data from the 2006 Swedish National Survey of Public Health were used for analyses. The total study population comprised a randomly selected representative sample of 26.305 men and 30.584 women aged 16–84 years. Harmful alcohol consumption was measured using a short version the Alcohol Use Disorders Identification Test (AUDIT), developed and recommended by the World Health Organisation. Low institutional trust was defined based on trust in ten main welfare institutions in Sweden.

**Results:**

Independent of age, country of birth and socioeconomic circumstances, low institutional trust was associated with increased likelihood of harmful alcohol consumption (OR (men) = 1.52, 95% CI 1.34–1.70) and (OR (women) = 1.50, 95% CI 1.35–1.66). This association was marginally altered after adjustment for interpersonal trust.

**Conclusion:**

Findings of the present study show that lack of trust in institutions is associated with increased likelihood of harmful alcohol consumption. We hope that findings in the present study will inspire similar studies in other contexts and contribute to more knowledge on the association between institutional trust and lifestyle patterns. This evidence may contribute to policies and strategies related to alcohol consumption.

## Background

Social capital entails civic engagement, social participation, trust in other people, trust in the formal institutions of society and generalized reciprocity[1]. Some authors have studied social capital as contextual characteristics of society [2,3], while others have investigated it from a micro-level perspective. Thus social capital in previous studies entails both social relations in the local environment and trust between individuals [4,5]. Social capital has often been operationalized as social participation and trust [1], but these two core components of social capital are not strongly correlated [6]. Trust includes the expectation that an individual or institution will act competently, fairly, openly, and with concern [7,8].

In the literature there has been an extensive debate whether values, such as trust, should be part of the definition of social capital or not. While for example, Woolcook [9] considers trust as important in its own right, he sees it as an outcome of social capital and therefore suggests excluding trust from the definition. On the other hand, Fukuyama [5] stresses the role of trust as a core component of social capital. According to Fukuyama, a nation's well-being, as well as its ability to compete, is dependent on the level of trust inherent in society [5].

Trust can be divided into vertical trust in the institutions of society (institutional trust) or horizontal trust (generalized trust in other people) [1]. Institutional trust, which is related to formal networks, is concerned with trust in the formal system, e.g. the political, tax or judicial system; whereas generalised trust can be characterised as trust in other people and is related to informal participation [10]. It is important to distinguish the different dimensions of trust, as they are not always related. For example, it is possible to trust people in general, but at the same time, mistrust the formal system. As Putnam [2] expresses it: 'One could easily trust one's neighbour and distrust city hall, or vice versa' (p. 137).

Institutional or vertical trust hence concerns the trust of the citizens in the institutions, especially the public institutions of society [11]. It should be noted that when we speak of trust in institutions (vertical trust) we mean something that is primarily a concept used to denote social relations *between *individuals and groups of individuals, and, on the other hand, institutions, not a characteristic of individuals. When we speak of generalized (horizontal) trust in other people we also essentially refer to a social construct. However, the only way to directly measure these constructs is to ask individuals in interviews or surveys.

Generalized trust in other individuals, as a measure of social capital, has been associated with good health and longevity in a number of studies [12-18]. In contrast, institutional trust in relation to health has largely been ignored in social capital research, in spite of the fact that institutional trust is believed to be crucial for modern societies [19]. At societal level, there might be positive health effects when healthy norms are spread and adopted in society, and social control over deviant behaviour [15]. For instance, high levels of trust in society can facilitate faster and wider diffusion of information, which may in turn promote healthier behaviours [20] and control unhealthy behaviours, such as smoking and alcohol abuse [21-24]. In contrast, low levels of trust in society may make the diffusion of information and the introduction of preventive policies concerning health behaviour more difficult.

Sweden is a welfare state with a long history of citizens' high level of trust in institutions [25]. However, it is well known that in many Western countries, including Sweden, there are increasing feelings of resentment towards the political systems. For example trust in politicians and the institutions of government has been shown to be declining continuously during the past decades in Sweden [26].

It is plausible that a trend towards decreasing trust in society may contribute to lack of cohesiveness in society and lack of responsible actions, which may ultimately promote unhealthy lifestyle behaviors, such as higher level of alcohol consumption and alcohol abuse. Alcohol consumption is important to investigate because it is one of the severe threats to public health. Over-consumption of alcohol beverages ranks 3^rd ^among the top causes of disability and substantially contribute to violence, accidents and premature death [27]. Alcohol consumption in Sweden has increased markedly during recent years, from 8.4 in 1996 to 10.2 liters per person in 2005 [28], and has been expected to lead to and increase alcohol related problems [29]. The core components of social capital, i.e. social participation and trust, may plausibly affect alcohol consumption in different ways. Social participation may increase or decrease alcohol consumption depending on the prevailing alcohol consumption patterns of the social group in which the individual participates. On the other hand, low trust is more likely to increase alcohol consumption for psychological reasons, due to the fact that poor mental health is partly related to low trust [6]. In a previous study in one region in Sweden, social participation was not statistically significantly associated with harmful alcohol consumption. On the contrary, there was a statistically significant association between low generalized (horizontal) interpersonal trust and high alcohol consumption among men but not among women [22]. Unfortunately institutional trust was not investigated in this previous study.

In this study we sought to investigate the association between alcohol consumption and institutional trust, while taking into account interpersonal trust.

It is worth noting that the level of alcohol consumption might be influenced by economic and social conditions including social capital, but also by laws and restrictions imposed by the government and the national parliament (in Sweden the *Riksdag*). More specifically, trust in institutions may be associated with alcohol consumption in several ways. In Sweden the National Board on Health and Welfare (*Socialstyrelsen*) is a governmental organization which formulates recommendations concerning different health related behaviours and other health risks. We believe that people with low trust in the institutions of society in general and *Socialstyrelsen *in particular may plausibly be less likely to follow such recommendations. Alcohol consumption has constituted a significant public health topic in Sweden and has been influenced by political decisions during the past century. The national parliament in Sweden (the *Riksdag*) has previously passed several laws and restrictions in order to restrict the availability of alcohol and thereby limit alcohol consumption. In spite the fact that Sweden is a European Community member still has an exception to keep the alcohol vending state monopoly. Thus lower political trust (an aspect of trust in institutions) in politicians, or in the *Riksdag *as an institution, may be associated with high alcohol consumption, and the direction of causality may go in both directions. Furthermore, trust in other institutions, for instance the entire health care system, may also be associated with alcohol consumption. This is because the public institutions in Sweden are consistent and coherent in the way they view aspects such as high alcohol consumption.

Thus a hypothesis that lack of institutional trust is associated with harmful alcohol consumption seems highly plausible. As far as we know the way in which trust in welfare institutions may influence patterns of alcohol consumption has not previously been empirically evaluated. The main aim of the present study was to analyze the association between institutional trust and alcohol consumption, while taking into account potential confounders.

## Methods

### Study population

The analyses were based on the 2006 Swedish National Public Health Survey, which was carried out by Statistics Sweden, in collaboration with a number of various health care regions and districts in Sweden and coordinated by the Swedish National Institute of Public Health. The total study population comprised a randomly selected sample of 56.889 individuals (26.305 men and 30.584 women) aged 16–84 years. The response rate was 60.1%. Detailed information on the study population and data collection is published elsewhere [30].

### Collection of data

Data was collected within a three-month period during spring 2006 and was based on a postal self-administered questionnaire linked to registry data from Statistics Sweden. Respondents were assured confidentiality and informed about data linkage with registry data. Data from the completed questionnaire were de-identified and controlled for errors, inconsistencies and internal missing data [31]. The present study was approved by the Department of Data Inspection, the Research Ethical Committee at the Swedish National Board of Health and Welfare (20031208) and the Stockholm Regional Ethics Committee (DNR 2005/1146-31).

### Main outcome

#### Alcohol consumption

The Alcohol Use Disorders Identification Test (AUDIT) is a 10-item self-report questionnaire, developed and recommended by the World Health Organisation, used to identify people whose alcohol consumption has become hazardous or harmful to their health [32]. The AUDIT's 10 items cover the three conceptual domains of consumption, dependence symptoms, and alcohol-related consequences that were intended to parallel the World Health Organization concepts of hazardous drinking, alcohol dependence, and alcohol-related harm [32]. Since AUDIT is sensitive, not only to severe alcohol problems, but also to hazardous drinking, it is particularly suitable for studies in the general population where prevalence of alcohol problems is lower than in clinical samples [33].

In this study harmful alcohol consumption is categorized based on the three consumption items of the Swedish version of AUDIT [33]. The Swedish version of AUDIT has been shown to have satisfactory internal and test-retest reliability [33]. Several previous studies have implicated that the three AUDIT items (sometimes discussed as AUDIT-C) is approximately equal in accuracy to the full AUDIT and can be employed as a stand-alone screening measure when time or other resources do not permit administration of the full AUDIT [34-38]. The three items includes; i) *"How often have you drunk alcohol in the past 12 months?" *Response options were; never (0 points); monthly or less (1 point); 2 to 4 times a month (2 points); 2 to 3 times a week (3 points); 4 times a week or more (4 points), (ii) *"How many glasses containing alcohol do you have on a typical day when you are drinking?" *(One drink is equivalent to to 5–8 cl of wine or 4 cl of alcoholic liquor, e.g., whisky). Response options were; 1 to 2 drinks (0 points); 3 to 4 drinks (1 point); 5 to 6 drinks (2 points); 7 to 9 drinks (3 points); or 10 or more drinks (4 points), (iii) *"How often do you have six drinks or more at one occasion?" *Response options were; never (0 points); less than monthly (1 point); monthly (2 points); weekly (3 points); or daily or almost daily (4 points). Hence, each item scored from 0 to 4 points with a maximum score of 12. Dichotomization of this score using well-established cut-off points has been suggested to identify hazardous or harmful habits[39]. In the present study, the cut-off for harmful alcohol consumption was set at 5 points for men and at 4 points for women [36]. This gender specific cut-off score has previously been recommended [40]. This is because women's metabolism system breaks down alcohol slower than men's thus often showing a higher blood-alcohol level than men's after consuming the same amount of alcohol consumed per kg body weight. Additionally the risks for medical alcohol-related harm, e.g. liver cirrhosis and cognitive disorder, are higher for women than for men [41].

### Main determinant

*Institutional trust *measures the vertical dimension of trust and reflects the person's perception of trust in welfare-state institutions, based on the ten most common welfare institutions in Sweden. These items have previously been used to measure institutional and political trust in Sweden [25], and other countries [42]. Institutional trust was measured based on the question; "How much trust do you have in the following institutions in society?" a) health care, b) school system, c) social welfare services, d) labor office, e) social insurance office, f) police, g) court of law, h) parliament, i) politicians at county council level and j) politicians at municipal level. Response options were; "Very high", "Fairly high", "Low", "No trust at all" and "No opinion". Because the internal consistency reliability was high (0.84), we constructed an index of institutional trust by summing up trust from all these ten institutions. Institutional trust was categorized as; (i) "Very high" (very high or fairly high trust in all ten institutions), (ii) "Moderately high" (low or no trust in 1–2 institutions), (iii) "Moderately low" (low or no trust in 3–5 institutions), (iiii) "Very low" (low or no trust in 6–10 institutions). The response "No opinion" was recorded as missing.

### Covariates

*Age *was categorized into 4 age groups: 16–29, 30–44, 46–64 and 65–84 years.

*Country of birth *was categorized as (i) Sweden, (ii) other OECD countries (other Nordic countries, Europe) or (ii) other countries (Africa, Asia, Latin America, Middle East).

*Educational attainment *was categorized into three levels (based on the highest registered examination level passed); (i) low (nine years compulsory school or less), (ii) intermediate (upper secondary school or less), (iii) high (university/college level).

*Financial stress *was recorded present if respondents gave "yes" answer to the question; "Have you during the last 12 months had difficulties paying ordinary bills (such as food, rent, etc.)?"

*Employment status *was categorized as (i) "employed", (ii) "other economically inactive" (e.g., students, sick-leave absence or maternity leave), (iii) "unemployed".

*Interpersonal trust *measures the horizontal dimension of trust and reflects the person's perception of trust in other people, and has been used in previous US [15] and Swedish studies [22]. Low interpersonal trust was recorded present if the respondents gave "no" answer to the question "Do you think that other people can be trusted in general?"

### Data analyses

Using SAS, version 9.1.3 we conducted logistic regression analyses to estimate the associations of alcohol consumption with institutional trust and other covariates. Prevalence (%) of harmful alcohol consumption, institutional trust, demographic, socioeconomic, and interpersonal trust variables were calculated (Table 1). Crude odds ratios and 95 % confidence intervals (OR, 95 %) were calculated in order to analyze associations between demographic, socioeconomic, interpersonal, institutional trust and high levels of alcohol consumption (Table 2). Three multiple logistic regression models adjusting for potential confounders (age, country of birth, education, employment status, financial stress, and interpersonal trust) were run to ascertain independent associations with alcohol consumption (Table 3). We found a statistically significant correlation between institutional and interpersonal trust (r = 0.19, P < 0.01). Therefore we further run the analyses on the association between institutional trust and alcohol consumption while stratifying on interpersonal trust (Table 4). All variables were simultaneously entered in the regression model. Analyses were conducted for men and women separately because of known gender differences in health and determinants of health [43].

**Table 1:** Prevalence (%) of harmful alcohol consumption, institutional trust, demographic, socioeconomic, and interpersonal trust variables (the Swedish National Public Health Survey 2006).

	Men (*N *= 26.305)	Women (*N *= 30.584)
	
	% (n)	% (n)
**Harmful alcohol consumption**.		
No	70.6 % (18442)	75.7 % (22975)
Yes	29.5% (7699)	8.5 % (7383)
(Missing)	(164)	(226)
		
**Institutional trust**		
Very high (high trust in all institutions)	12.1 % (3090)	16.4 % (4836)
Moderately high (lack of trust in 1–2 institutions)	18.1 % (4614)	23.4 % (6884)
Moderately low (lack of trust in 3–5 institutions)	34.9 % (8880)	34.8 % (10253)
Very low (lack of trust in 6–10 institutions)	34.9 % (8875)	25.5 % (7502)
	(846)	(1109)
		
**Age (years)**	8.1 % (4761)	19.5 % (5973)
16–29	22.7 % (5967)	23.8 % (7288)
30–44	32.9 % (8647)	30.9 % (9640)
45–64	26.3 % (6930)	25.7 % (7863)
65–84	(0)	(0)
(Missing)		
		
**Country of birth**	91.4 % (24042)	90.2 % (27596)
Sweden	5.6 % (1565)	7.2 % (2193)
OECD (Other Nordic, Europe, North America)	2.7 % (698)	2.6 % (795)
Other (Asia, Latin America, Africa)	(0)	(0)
(Missing)		
		
**Educational level**	13.4 % (3167)	17.5 % (4796)
High	32.1 % (7585)	31.5 % (8267)
Intermediate	54.5 % (12888)	51.0 % (13978)
Low	(2665)	(3183)
(Missing)		
		
**Financial stress during the past year**	85.7 % (22314)	81.2 % (24876)
No	14.3 % (3725)	17.8 % (5373)
Yes	(266)	(335)
(Missing)		
		
**Employment status**	52.6 % (13328)	48.0 % (14027)
Employed	42.6 % (10799)	47.1 % (13772)
Other activities (students, parental leave etc.)	4.8 % (1202)	4.9 % (1438)
Unemployed	(976)	(1347)
(Missing)		
		
**Interpersonal Trust**		
High	75.4 % (19332)	75.6 % (22538)
Low	24.6 % (6319)	24.4 % (7276)
(Missing)	(654)	(770)

**Table 2:** Prevalence's (%) and crude odds ratios (OR) and 95 % confidence intervals (CI) of hazardous alcohol consumption in relation to institutional trust, demographic, socioeconomic, and interpersonal trust variables (the Swedish National Public Health Survey 2006).

	Men (*N *= 26.305)		Women (*N *= 30.584)	
	
	Percent	OR (95% CI)	Percent	Percent
**Institutional trust**				
Very high (high trust in all institutions)	20.0 %	1.00	15.8 %	1.00
Moderately high (lack of trust in 1–2 institutions)	26.7 %	1.44 (1.29–1.61)	24.0 %	1.68 (1.53–1.85)
Moderately low (lack of trust in 3–5 institutions)	30.4%	1.73 (1.57–1.91)	26.6 %	1.93 (1.77–2.10)
Very low (lack of trust in 6–10 institutions)	34.0 %	2.04 (1.86–2.25)	28.3 %	2.10 (1.92–2.31)
	(967)		(1264)	

**Age (years)**				
16–29	48.4 %	1.00	47.3 %	1.00
30–44	37.3 %	0.64 (0.59–0.69)	26.6 %	0.40 (0.38–0.44)
45–64	28.4 %	0.42 (0.39–0.46)	22.5 %	0.32 (0.30–0.35)
65–84	10.9 %	0.13 (0.12–0.14)	6.7 %	0.08 (0.07–0.09)
(Missing)	(164)		(226)	

**Country of birth**				
Sweden	30.9 %	1.00	25.2 %	1.00
OECD (Other Nordic, Europe, North America)	26.0 %	0.82 (0.73–0.92)	18.3 %	0.67 (0.60–0.75)
Other (Asia, Latin America, Africa)	15.1 %	0.41 (0.33–0.51)	11.8 %	0.40 (0.32–0.50)
(Missing)	(164)		(226)	

**Educational level**				
High	28.3 %	1.00	25.8 %	1.00
Intermediate	38.8 %	1.41 (1.29–1.54)	34.4 %	1.51 (1.40–1.64)
Low	31.0 %	0.88 (1.29–1.55)	25.8 %	0.81 (0.75–0.87)
(Missing)	(2784)		(3323)	

**Financial stress during the past year**				
No	27.9 %	1.00	22.3 %	1.00
Yes	39.6%	1.69 (1.58–1.82)	33.7 %	1.76 (1.65–1.88)
(Missing)	(389)		(505)	

**Employment status**				
Employed	36.7 %	1.00	29.7 %	1.00
Other activities (students, parental leave etc.)	19.2 %	0.41 (0.39–0.43)	18.1 %	0.54 (0.51–0.57)
Unemployed	39.4 %	1.12 (0.99–1.27)	35.2 %	1.31 (1.17–1.47)
(Missing)	(1113)		(1544)	

**Interpersonal Trust**				
High	28.59 %	1.00	23.2 %	1.00
Low	33.2 %	1.24 (1.17–1.32)	28.8 %	1.34 (1.26–1.42)
(Missing)	(771)		(307)	

**Table 3:** Age adjusted and multivariate adjusted odds ratios (OR) and 95 % confidence intervals (CI) of hazardous alcohol consumption in relation to institutional and interpersonal trust (the Swedish National Public Health Survey 2006).

Institutional trust	**Model 1**	**Model 2**	**Model 3**
	
	**OR **(95 % CI)	**OR **(95 % CI)	**OR **(95 % CI)
**Men **(*N *= 18.558)			

**Institutional trust**			
Very high (high trust in all institutions)	1.00	1.00	1.00
Moderately high (lack of trust in 1–2 institutions)	1.29 (1.15–1.44)	1.18 (1.04–1.33)	1.18 (1.04–1.33)
Moderately low (lack of trust in 3–5 institutions)	1.58 (1.42–1.75)	1.40 (1.25–1.56)	1.39 (1.24–1.55)
Very low (lack of trust in 6–10 institutions)	1.76 (1.58–1.95)	1.52 (1.36–1.70)	1.50 (1.34–1.68)
			
**Interpersonal Trust**			
High (reference category)	1.00	1.00	1.00
Low	1.07 (1.01–1.40)	1.11 (1.04–1.19)	1.06 (0.98–1.13)

**Women **(*N *= 21.444)			

**Institutional trust**			
Very high (high trust in all institutions)	1.00	1.00	1.00
Moderately high (lack of trust in 1–2 institutions)	1.35 (1.22–1.49)	1.27 (1.14–1.41)	1.27 (1.14–1.42)
Moderately low (lack of trust in 3–5 institutions)	1.58 (1.44–1.74)	1.43 (1.30–1.58)	1.43 (1.34–1.58)
Very low (lack of trust in 6–10 institutions)	1.64 (1.49–1.81)	1.50 (1.35–1.66)	1.48 (1.34–1.65)
			
**Interpersonal Trust**			
High (reference category)	1.00	1.00	1.00
Low	1.04 (0.98–1.11)	1.09 (1.02–1.17)	1.05 (0.98–1.13)

Model 1. Adjusted for age

Model 2. Simultaneously adjusted for age, country of birth, educational level, financial stress and employment status Model

3. Simultaneously adjusted for age, country of birth, educational level, financial stress and employment status, institutional and interpersonal trust

**Table 4:** Association between institutional trust and harmful alcohol consumption after stratifying on interpersonal trust (Swedish National Public Health Survey 2006).

**Institutional trust**	**Low interpersonal trust**	**High interpersonal trust**
	
	**OR **(95 % CI)	**OR **(95 % CI)
**Men**	N = 6319	N = 19332

**Institutional trust**		
Very high (high trust in all institutions)	1.00	1.00
Moderately high (lack of trust in 1–2 institutions)	1.29 (0.99–1.70)	1.45 (1.29–1.64)
Moderately low (lack of trust in 3–5 institutions)	1.51 (1.17–1.94)	1.74 (1.56–1.93)
Very low (lack of trust in 6–10 institutions)	1.78 (1.40–2.27)	2.00 (1.79–2.24)

**Women**	N = 7276	N = 22538

**Institutional trust**		
Very high (high trust in all institutions)	1.00	1.00
Moderately high (lack of trust in 1–2 institutions)	1.39 (1.11–1.74)	1.72 (1.55–1.92)
Moderately low (lack of trust in 3–5 institutions)	1.60 (1.30–1.97)	1.95 (1.76–2.15)
Very low (lack of trust in 6–10 institutions)	1.64 (1.33–2.01)	2.09 (1.88–2.33)

Simultaneously adjusted for age, country of birth, educational level, financial stress and employment status

## Results

Men were more likely than women to report harmful alcohol consumption (30 % vs. 24 %). Women were more likely to report the highest level of institutional trust (16 %), to experience financial stress (18 %) than men (12 % and 14 % respectively). Men were more likely to have a low level of education (55 %) than women (51 %). There were no significant gender differences in the distribution of other study variables (Table 1).

Prevalence of harmful alcohol consumption decreased with older age among both men and women. Socioeconomic disadvantage (financial stress and being unemployed) and low interpersonal trust were associated with harmful alcohol consumption, while low educational level was not. The likelihood of harmful alcohol consumption differed significantly in a graded fashion in relation to levels of high institutional trust. The crude odds ratios for the "lowest institutional trust" category were 2.04 (1.86–2.25) and 2.20 (1.92–2.31) for men and women respectively, as compared with the very high trust category (Table 2).

The odds ratios were considerably reduced after further adjustment for age, country of birth, educational level, financial stress and employment status in the models, reducing the odds ratios of the "lowest institutional trust" category to 1.52 (1.36–1.70) and 1.50 (1.35–1.66) for men and women, respectively. These results remained statistically significant even after further adjustment for interpersonal trust (Table 3). Low interpersonal trust was independently associated with hazardous alcohol consumption after adjustment for age, country of birth, educational level, financial stress and employment status in the models (OR for men 1.10 (1.04–1.19) and OR for women 1.09 (1.02–1.17)) (Table 3). The association between low interpersonal trust and harmful alcohol consumption was not statistically significant after further adjustment for institutional trust (Table 3). In the stratified analyses in Table 4 it is demonstrated that all levels of lower institutional trust were associated with harmful alcohol consumption even in presence of high interpersonal trust (Table 4).

## Discussion

The results of the present study show that, independent of age, country of birth, socioeconomic circumstances, low institutional trust was associated with an increased risk of harmful alcohol consumption of 50 percent fold among men and of 48 percent fold among women. We found a statistically significant graded association between harmful alcohol consumption and low levels of institutional trust even in presence of high interpersonal trust.

In spite of the fact that trust in institutions is argued to be important in highly democratic modern societies as that of Sweden [19] the existing literature has focused on interpersonal trust. As far as we know this is the first study where both institutional and interpersonal trust are studied showing independent associations of low institutional trust with harmful alcohol consumption, even after adjusting and stratifying for interpersonal trust. We considered interpersonal trust in the analyses. This is because trust is perceived to be a complex and multi-dimensional phenomenon, consisting of a mix of trust in strong ties, weak ties, institutions, and personal traits including a possible psychological component of paranoia or aggressiveness [44]. Thus trust may be considered to be a less objective measure of real trust levels for institutions. However our analyses stratifying for interpersonal trust do not seem to support this possibility. We found that low levels of institutional trust even in presence of high interpersonal trust increased the likelihood of harmful alcohol consumption.

Additionally we did not find statistically significant correlation between institutional trust and psychological well-being (measured by GHQ-12) (r = 0.09). Nevertheless, even after further adjustment for psychological distress, social support and social participation, the association between low institutional trust and harmful alcohol consumption remained statistically significant. It seems likely that institutional trust may comprise a structural component of how institutions are perceived in general. It should be noted that institutional trust does not seem to be correlated with indicators of social capital at individual level. Further more in the present paper we found a weak and not statistically significant correlation of institutional trust with instrumental social support (r = 0.08), with emotional social support (r = 0.07) nor with social participation (r = 0.03). These results are similar to other previous findings [6].

Sweden is a welfare state with a long history of trust and investment in institutions, playing a substantial role for the well-being of inhabitants. It is plausible that low trust in institutions may contribute to lack of cohesiveness in society and a lack of responsible actions, which may ultimately promote unhealthy lifestyle behaviors. This is based on one of Fukuyama's arguments that a society's well-being and actions depend on the level of trust [5]. High trust in institutions' may on the other hand increase the inclination to follow advice and health information, and to take part in prevention. In fact, it is surprising that the association between trust in institutions and different aspects of public health have been studied to such a limited extent, considering the fact that macro-level politics and policies are generally regarded as crucial for population health [41,44]. High political trust in the government, the Riksdag and its politicians may lead to a higher propensity to follow the rules and regulations decided by these authorities, also in the alcohol policy area. High institutional trust in the national Board on Health and Welfare may also lead to a higher likelihood of following the information and recommendations concerning for instance alcohol consumption given by this public institution. Furthermore, high institutional trust in the healthcare system may lead to a higher inclination to follow advice and recommendations from the health care system centrally as well as from specific physicians and nurses concerning for instance alcohol consumption, and the list could be made even longer by adding other public institutions.

The gender differences observed in prevalence of institutional trust are worthy noting. A larger proportion of men (35%) than women (26%) reported very low institutional trust. We also observed that the likelihood of harmful alcohol consumption in relation to very low trust was larger for men (50%) than for women (48%). These gender differences are not similar to results from the Australian study which demonstrated that a high social capital index, including trust (both interpersonal and institutional), had larger effects on women's self-rated health than men's [17]. The authors suggested that women may benefit more than men from higher levels of area social capital. However, in the case of harmful alcohol consumption, high levels of social capital (high trust) seem to be more protective for men than for women. From a public health policy perspective, there is a need to analyze gender differences in future studies related to social capital and health.

Results of the present study should be interpreted considering its strengths and limitations. First, the cross-sectional design of this study makes it difficult to draw conclusions about causal associations between low institutional trust and harmful alcohol consumption. Also the changing patterns of trust and alcohol consumption habits could not be captured. However, results in the present study show strong associations between institutional trust and a validated measure of harmful alcohol consumption [34] which persisted even after adjusting for potential confounders using a large data set that represents the general population of Sweden. Second, the self-reported prevalence of harmful alcohol consumption in the present study might have been underestimated compared with data from sales statistics. Nevertheless, self-reported alcohol consumption is considered to be a valid measure of an underlying alcohol problem [45].

Third, the non-response rate of 37%, which included a large proportion of men, socially disadvantaged, and inhabitants in metropolitan areas is problematic. Unfortunately, we did not have information on the level of alcohol consumption and trust, however it is this sub-group which is usually at a high risk for harmful alcohol consumption [39]. Thus results presented here may actually be an under-estimation of the prevalence of harmful alcohol consumption and of the magnitude of true associations demonstrated in the present paper.

Fourth, our study does not evaluate associations from a lifecourse perspective. Norms and values, particularly in relation to harmful alcohol consumption, have been demonstrated to be formed in the socialization process during childhood and adolescence [46].

## Conclusion

The results from the present study show that lack of trust in institutions is associated with increased likelihood of harmful alcohol consumption. Public health policies should be designed with the consideration of the characteristics of relevant institutions and how these may impact on lifestyle patterns of individuals that include levels of alcohol consumption. We hope that findings in the present study will inspire similar studies in other contexts and contribute to more knowledge on the evidence of the association between institutional trust and lifestyle patterns. This evidence may be an important contribution to policies and strategies related to alcohol consumption.

## Competing interests

The authors declare that they have no competing interests.

## Authors' contributions

JA participated in the design of the study, was responsible for preparation of the dataset, performed the statistical analysis and drafted the manuscript. SPW conceived of the study, and participated in its design and coordination and helped to draft the manuscript. ML participated in the design of the study and coordination and helped to draft the manuscript.

## Pre-publication history

The pre-publication history for this paper can be accessed here:


